# Extracellular Vesicle-Associated mir-21 and mir-144 Are Markedly Elevated in Serum of Patients With Hepatocellular Carcinoma

**DOI:** 10.3389/fphys.2018.00930

**Published:** 2018-07-17

**Authors:** Chunwen Pu, Hui Huang, Zhidong Wang, Wei Zou, Yuecai Lv, Zhiyuan Zhou, Qiqi Zhang, Liang Qiao, Fei Wu, Shujuan Shao

**Affiliations:** ^1^Department of Biobank, The Affiliated Sixth People’s Hospital of Dalian Medical University, Dalian, China; ^2^The Affiliated Zhongshan Hospital of Dalian University, Dalian, China; ^3^College of Life Science, Liaoning Normal University, Dalian, China; ^4^Key Laboratory of Proteomics, Dalian Medical University, Dalian, China; ^5^The University of Sydney at Westmead Hospital, Westmead, NSW, Australia

**Keywords:** microRNA, extracellular vesicle, sequencing analysis, qRT-PCR, hepatocellular carcinoma

## Abstract

**Aim:** The aim of this study was to observe the possible change of microRNAs (miRNAs) in serum extracellular vesicles (EVs) from hepatocellular carcinoma (HCC) patients.

**Methods:** The serum EVs were purified from 17 healthy donors, 16 chronic hepatitis B (CHB) patients and 24 HCC patients. The sequenced microRNAs in the purified EVs were analyzed to obtain highly differentially expressed genes (DEGs). Finally, the expression pattern of DEGs was validated using qRT-PCR.

**Results:** We found that the expression of hsa-miR-21-5p and hsa-miR-144-3p were significantly higher in EVs and liver cancer tissues compared with serum and the distal liver tissues in HCC patients. The ratio of hsa-miR-144-3p/hsa-miR-21-5p was significantly decreased in the patients with CHB but significantly increased in patients with HCC developed from CHB (*P* < 0.05). Hsa-144-3p/hsa-miR-21-5p exhibited greater performance than alpha-fetoprotein (AUC 0.780, 95% CI 0.601–0.960, versus AUC 0.626, 95% CI 0.410–0.843) in ROC curve analysis.

**Conclusion:** Extracellular vesicle-associated hsa-miR-21-5p and hsa-miR-144-3p are markedly elevated in serum of patients with HCC. The potential role of these microRNAs in the pathogenesis of HCC is worth of further study.

## Introduction

Hepatocellular carcinoma (HCC) is the fifth most common cancer in men and the ninth most common cancer in women. HCC is the second most common cause of cancer-related death worldwide, approximately 746,000 deaths in 2012 (GLOBOCAN, 2012). In China, the incidence and mortality of liver cancer continuously increase in recent years. Currently, HCC has the worst prognosis among all major cancers, mainly due to the lack of early sensitive diagnostic markers.

Currently, the diagnosis of HCC mainly depends on the imaging and serum markers such as alpha-fetoprotein (AFP). AFP is the most commonly used biomarker for diagnosis of HCC in China (National Health and Family Planning Commission of the PRC, 2011), and elevated AFP protein levels are commonly used as serological markers of HCC. However, AFP is also elevated in the patients with non-cancerous liver disease such as hepatitis or cirrhosis (Bloomer et al., 1975), and AFP is also produced and released from injured liver tissue or other tissues (Nunez, 1994; Yin and Wang, 2003; Wong et al., 2015). In recent years, the number of the HCC patients with low-level of AFP or even AFP-negative has been increasing. In addition, the detection rate of the patients with small HCC (<2 cm) using AFP as a marker usually is very low. Thus, AFP is not a good biomarker for detection and diagnosis of HCC due to its low specificity and sensitivity (Sun, 2002). Thus, novel biomarkers with high specificity and sensitivity are urgently needed the early and accurate detection of HCC.

Liquid biopsy monitors circulating tumor cells (CTCs), DNA and extracellular vesicles (EVs) released into the blood which provides novel approaches for diagnosis of different diseases including HCC. The EVs are spherical or cup-shaped vesicles with a size of 30–100 nm (Kalluri, 2016). EVs consist of lipids, proteins, RNAs, and DNAs (Morelli et al., 2016), which mediate intercellular transmission of biologically active molecules. Accumulating evidences indicated that EVs are involved in many liver diseases including hepatitis C virus (HCV) infection (Longatti et al., 2015), hepatitis B virus (HBV) infection (Yang et al., 2017), HCC (Wei et al., 2015), liver fibrosis (Huang and Brigstock, 2012), cirrhosis (Witek et al., 2009), non-alcoholic fatty liver disease (NAFLD), and alcoholic liver disease (ALD) (Koeck et al., 2014). In addition, EV can be widely detected in human blood (Caby et al., 2005), urine (Pisitkun et al., 2004), saliva (Ogawa et al., 2011), bile (Masyuk et al., 2010), milk (Lässer et al., 2011), and amniotic fluid (Asea et al., 2008). Thus, investigation of the miRNA and protein in EVs may provide novel biomarkers for early and accurate diagnosis of HCC and other liver diseases.

In this study, we purified serum EVs from healthy donors and patients with CHB and HCC, and verified differentially expressed miRNAs to seek potential diagnostic biomarkers for HCC.

## Materials and Methods

### Patient Characteristics

A total of 57 subjects from January, 2015 to 2017 were recruited from the Affiliated Sixth People’s Hospital of Dalian Medical University. The samples in this study were obtained with informed consent. The study was approved by Ethics Committee of the Affiliated Sixth People’s Hospital of Dalian Medical University (Approval ID: 2017-001-003). Written informed consent was obtained from each participate before being enrolled into the study. Patient characteristics are shown in **Table 1**. The recruiters were divided into three groups: healthy donors (*n* = 17), CHB patients (*n* = 16), and HCC patients (*n* = 24). CHB and HCC were diagnosed according to the criteria of the Asian Pacific Association for the Study of the Liver. Healthy donors were individuals who were in healthy condition without detectable malignancy.

**Table 1:** Clinical characteristics according to the etiology.

Clinical parameter	Healthy donors (*n* = 17)	CHB (*n* = 16)	HCC (*n* = 24)
Age (year)	45.94 ± 6.49	47.06 ± 13.37	59.33 ± 8.18
Gender (M/F)	11/6	11/5	18/6
ALT (U/L)	19.39 ± 7.34	113.79 ± 92.49	113.52 ± 176.47
AST (U/L)	21.97 ± 5.37	60.64 ± 46.93	64.41 ± 78.52
TBiL (μmol/L)	16.61 ± 5.58	15.74 ± 5.79	24.83 ± 18.08
AFP (ng/ml)	/	6.25 ± 6.64	406.40 ± 1174.12
AFP > 100 ng/ml	*n* = 0	*n* = 0	*n* = 5
small HCC (<2 cm)	*n* = 0	*n* = 0	*n* = 9
CA19-9 (U/mL)	/	32.24 ± 40.94	28.98 ± 29.62
CEA (ng/mL)	/	1.52 ± 0.99	2.98 ± 1.99
HBsAg (positive/negative)	0/17	16/0	19/5


*“/” Represents no data.*

### Purification of Human Serum Extracellular Vesicles Using Exo-spin Extracellular Vesicle Purification Kit

Serum EVs of healthy donors, CHB and HCC patients were extracted and purified using Exo-spin EV Purification Kit. Cell debris was removed and EVs were purified from 500 μL serum according to the Exo-spin EV Purification kit (cell guidance system, EXO-2) instructions.

### Transmission Electron Microscopy

Extracellular vesicles were solubilized with 20 μL PBS and fixed with 4% paraformaldehyde. Copper mesh was infiltrated in the EV solution for 2 min, and excess PBS solution was blotted with filter paper. Copper mesh immersed in uranyl acetate solution for 2 min, and excess liquid was blotted with filter paper. At last, the copper mesh was placed under transmission electron microscopy to collect images.

### ZetaView Analysis

We measured the EV particle size and concentration using nanoparticle tracking analysis (NTA) with ZetaView PMX 110 (Particle Metrix, Meerbusch, Germany) and corresponding software ZetaView 8.04.02. Isolated EVs were diluted using 1X PBS buffer (Biological Industries, Israel) to measure the particle size and concentration. NTA measurement was recorded and analyzed at 11 positions. The ZetaView system was calibrated using 110 nm polystyrene particles. Temperature was maintained around 27.68°C. PH was around 7.4.

### Western Blotting Analysis

The cluster of differentiation 63 (CD63) and CD9 are often enriched in EVs. To validate the isolation of the EVs from the serums, western blotting analysis was performed using the mouse polyclonal anti-human (CD63) (Santa Cruz, sc-5275) and mouse polyclonal anti-human CD9 antibodies (Santa Cruz, sc-13118), purified EV pellets from the serums were washed in PBS and lyzed with the RIPA lysis buffer. Western blotting was followed by rabbit anti-mouse horseradish peroxidase (System Biosciences). The protein concentration was measured using the bicinchoninic acid kit according to the manufacturer’s instruction. The proteins were separated on 10% SDS-polyacrylamide gels and transferred to PVDF membranes. The membranes were blocked with 5% non-fat milk dissolved in TBS Tween-20 (50 mM Tris HCl, pH 7.6, 150 mM NaCl, 0.2% Tween-20) for 1 h and incubated with primary antibodies at 4°C overnight. The blottings were then incubated with appropriate IgG conjugated to horseradish peroxidase (1:5000).

### Quantitative Real-Time PCR of miRNAs

Two miRNAs were verified using the quantitative real-time PCR (qRT-PCR) method. Primers of miRNAs for qRT-PCR are hsa-miR-21-5p (CD201-0092, TIANGEN) and hsa-miR-144-3p (CD201-0011, TIANGEN). The experiment of qRT-PCR with an internal control U6 (CR-100-01, TIANGEN) was performed to validate the expression levels of two miRNAs by using the miRcute Plus miRNA qPCR Detection Kit (SYBR Green, FP411, TIANGEN) according to the manufacturer’s protocol. The experiments were carried out in triplicate with a total volume of 20 μL in a qRT-PCR instrument (TaKaRaDiceRealTime, TaKaRa), containing 10 μL of 2× miRcute Plus miRNA Premix (with SYBR and ROX), 1 μL of cDNA (500 ng), and 0.4 μL of forward and reverse primers (2 μmol/L). The qRT-PCR was programmed at 95°C for 15 min, followed by 40 cycles of 94°C for 20 s, and 60°C for 34 s. The expression level was calculated by the 2^-ΔΔC_T_^ method and subjected to statistical analysis.

## Results

### Purification and Validation of Serum Extracellular Vesicles From Human Peripheral Blood

**FIGURE 1:**
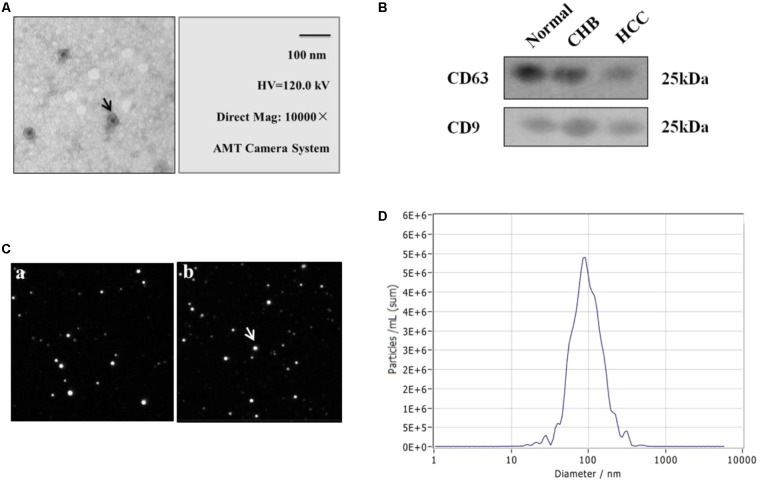
Identification and characterization of extracellular vesicles (EVs) of healthy donors and patients with CHB and HCC. **(A)** Transmission electron micrograph of purified EVs shows small vesicles with sizes ranging from 30 to 110 nm in diameter (bars 100 nm). Arrow represented EVs. **(B)** Western blotting analysis of EV-associated proteins CD9 and CD63 in purified EVs from normal individuals, CHB and HCC patients. **(C)** Morphology of EVs by ZetaView. **(a)** Extraction by ultracentrifugation. **(b)** Extraction by Exo-spin EV Purification Kit. Analysis parameters: Max Area: 1000, Min Area: 5, Min Brightness: 20. **(D)** Particle size and concentration distribution of EVs by ZetaView.

The peripheral blood was collected from individuals at the time of clinical consultation and processed as described in Section “Materials and Methods”. EVs were purified from 500 μL serum of healthy donors, CHB and HCC patients using EV purification kit. The isolated EVs were further examined with transmission electron microscopy, ZetaView and western blotting, which revealed a population of nano-vesicles with an approximate size of 100 nm and cup-shaped morphology typical of EVs (**Figures 1A,C**). The morphology of EVs extracted by ultracentrifugation and kits was complete and numerous (**Figures 1A–C**). In addition, western blotting analysis of protein extracts from the isolated EVs confirmed that the particles expressed characteristic EV markers CD9 and CD63 (**Figure 1B**). Besides, the concentration of particles was five million per milliliter of PBS (**Figure 1D**). The above results show that we obtained EVs with high purity and concentration.

### High Expression of miR-21-5p and miR-144-3p in Extracellular Vesicles and Liver Cancer Tissues of Patients With HCC

**FIGURE 2:**
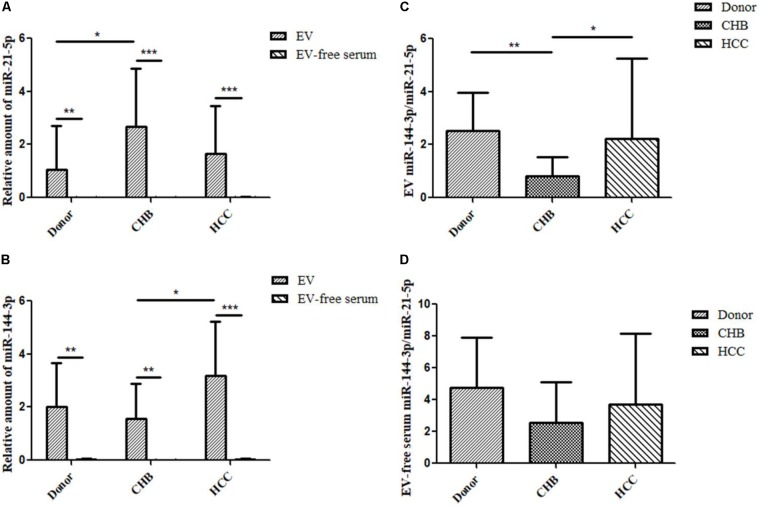
Relative expression of miR-21-5p and miR-144-3p of EVs were higher in patients with HCC. **(A)** The relative expression of miR-21-5p of EVs and EV-free serum in healthy donors and patients with CHB and HCC by qRT-PCR (Normalized by U6). **(B)** The relative expression of miR-144-3p of EVs and EV-free serum in healthy donors and patients with CHB and HCC by qRT-PCR (Normalized by U6). **(C)** The ratio of miR-144-3p/miR-21-5p in EVs of healthy donors and patients with CHB and HCC. **(D)** The ratio of miR-144-3p/miR-21-5p in EV-free serum of healthy donors and patients with CHB and HCC. ^∗^*P* < 0.05, ^∗∗^*P* < 0.01, and ^∗∗∗^*P* < 0.001.

Extracellular vesicles contain a variety of miRNAs among which miR-21 is an important marker identified in several studies of liver diseases. Compared with healthy donors, miR-21 is highly expressed in HCC patients (Wang et al., 2014). MiR-144 is a miRNA that plays a role as a tumor suppressor in many cancers. However, there are few studies on the expression of miR-144 in serum EVs of HCC. In order to investigate the expression of miR-21-5p and miR-144-3p of EVs in healthy donors and patients with CHB and HCC, we performed qRT-PCR experiments to validate the changes in the expression of miR-21-5p and miR-144-3p of EVs in healthy donors and patients with CHB and HCC. The results showed that the expression of miR-21-5p and miR-144-3p in EVs was significantly higher than that in EV-free serum (**Figures 2A,B**). This indicates that miR-21-5p and miR-144-3p were significantly enriched in EVs. Besides, the data obtained from RNA-seq in early period was consistent with the result of qRT-PCR in which that the expression of hsa-miR-21-5p of EVs in patients with HCC was higher than that in healthy donor group. Moreover, the expression of miR-21-5p in patients with CHB was significantly higher than that in healthy donor people. In other ways, the expression of miR-21-5p was highest in EVs of patients with CHB, followed by HCC group, and which was lowest in healthy donor group (**Figure 2A**). On the other side, the expression of miR-21-5p in EV-free serum in patients with HCC, CHB and healthy donors was decreased in turn (**Figure 2A**). In addition, the expression of miR-144-3p was highest in EVs of patients with HCC, followed by healthy donor group, and which was lowest in CHB group seen from **Figure 2B**, which was consistent with the result of RNA-seq in early period. Moreover, the expression of miR-144-3p in patients with HCC was significantly higher than that in patients with CHB. The expression of miR-144-3p in EV-free serum in patients with HCC, CHB and healthy donors was decreased in turn (**Figure 2B**).

**FIGURE 3:**
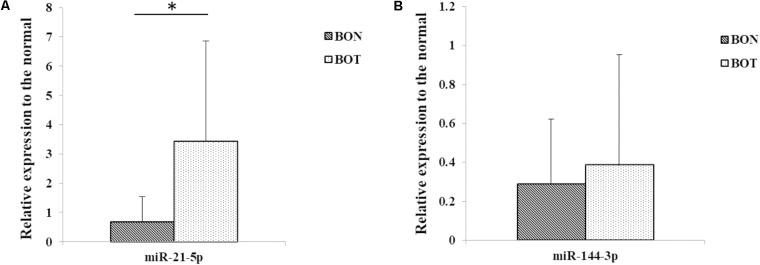
Relative expression of miR-21-5p and miR-144-3p of liver cancer tissues were higher in patients with HCC. **(A)** The relative expression of miR-21-5p in distal tissues of hepatic carcinoma (BON) and hepatocellular carcinoma (BOT) (Normalized by U6). **(B)** The relative expression of miR-144-3p in distal tissues of hepatic carcinoma (BON) and hepatocellular carcinoma (BOT) (Normalized by U6). ^∗^*P* < 0.05.

In view of the found of high expression of miR-21-5p and miR-144-3p in serum EVs of HCC, we also performed qRT-PCR experiments in hepatocellular carcinoma (BOT) and the distal tissues of hepatic carcinoma (BON). The results showed that the expression of miR-21-5p in BOT was significantly higher than that in BON (**Figure 3A**), which was consistent with the performance of miR-21-5p in EVs. In addition, compared with BON group, miR-144-3p was highly expressed in BOT (**Figure 3B**), which was consistent with expression in EVs. The above results suggest that miR-21-5p and miR-144-3p were highly expressed in patients with HCC and may become potential biomarkers for HCC.

We have found that the expression levels of miR-21-5p and miR-144-3p in EVs of patients with HCC were higher than that in healthy donor group, but the significance of their relationship in clinical diagnosis is not yet clear. In order to clarify the relationship between miR-21-5p and miR-144-3p, we count the ratio of miR-144-3p/miR-21-5p. The results showed that the ratio of miR-144-3p/miR-21-5p in EVs of the three groups was higher than that in EV-free serum (**Figures 2C,D**). Among them, the ratio of miR-144-3p/miR-21-5p of EVs in patients with CHB was significantly reduced compared with healthy donor group (*P* < 0.01) (**Figure 2C**). Besides, the ratio of miR-144-3p/miR-21-5p was significantly increased in the HCC group compared with patients with CHB (*P* < 0.05) (**Figure 2C**). The ratio of miR-144-3p/miR-21-5p in EV-free serum showed the same trend as that in EVs (**Figure 2D**). It can be seen that with the development of HCC, the ratio of miR-144-3p/miR-21-5p in EVs shows a trend of first decrease and then increase and which can be used as an indicator of CHB and HCC diagnosis.

### The ROC Curve Analysis of miR-144-3p/miR-21-5p and AFP in HCC

**FIGURE 4:**
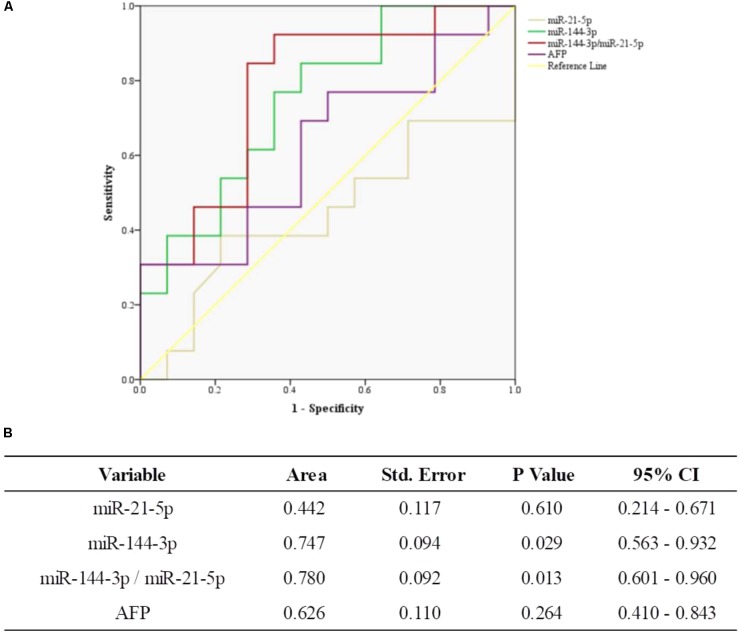
The general diagnostic efficiency of miR-144-3p/miR-21-5p and AFP for HCC. **(A)** ROC curves of miR-21-5p, miR-144-3p, and miR-144-3p/miR-21-5p for patients with HCC versus AFP. **(B)** The area under the ROC curve.

In this study, we further compared miR-144-3p/miR-21-5p and AFP in HCC patients. The ROC curves showed that miR-144-3p had greater performance than AFP (AUC 0.747, 95% CI 0.563–0.932 versus AUC 0.626, 95% CI 0.410–0.843, **Figures 4A,B**). Meanwhile, miR-144-3p/miR-21-5p had greater performance than AFP (AUC 0.780, 95% CI 0.601–0.960, versus AUC 0.626, 95% CI 0.410–0.843, **Figures 4A,B**). The above results suggested that miR-144-3p/miR-21-5p in EVs remains its diagnostic efficiency in HCC patients. All the results of ROC curves were presented in **Figure 4**.

## Discussion

Hepatocellular carcinoma is the second most common cause of death from cancer worldwide, which has the worst prognosis among all major cancers, largely due to the lack of sensitive diagnostic markers. Currently, the clinical diagnosis of liver cancer mainly depends on imaging and serum markers such as AFP. However, AFP has limitations in the detection of liver cancer due to its lack of tumor specificity and sensitivity (Nunez, 1994; Sun, 2002; Yin and Wang, 2003; Wong et al., 2015). In this study, the average level of AFP in serum of twenty-four HCC patients is about 406 ng/mL, but only 5/24 of HCC patients who’s AFP exceed the detection range. Therefore, AFP is not sufficient for early and accurate diagnosis of HCC. The aim of this study was to observe the possible change of miRNAs in serum EVs from HCC patients.

Extracellular vesicles opened up new opportunities of new method to diagnose cancer. EVs are rich of lipids, proteins, RNAs, and DNAs (Morelli et al., 2016) and mediate intercellular transmission of information by transferring different biologically active molecules. The recent founding that miRNAs circulate in a stable form in blood (Xiao et al., 2013), suggest that circulating miRNAs can serve as biomarkers, as well as function as mediators of disease, and protection from disease. In this study, we isolated and purified EVs from 500 μL serum of healthy donors and patients with CHB and HCC by ultracentrifugation and kit. We found that miR-21-5p and miR-144-3p were significantly enriched in EVs, while which were less and no significant change in EV-free serum, which means that serum EVs are highly enriched in miRNAs and EVs significantly increased the sensitivity of miRNA detection in serum. For instance, Skog et al. (2008) found that miR-21 was 40-fold higher in glioblastoma serum EVs than in EV-depleted fractions. Li et al. (2014) found that there was an almost fourfold increase in miR-144 precursor in the EV pellet, but there was no change in plasma microparticle (50–400 nM) numbers or their miR-144 content, suggesting that the detection of miRNAs in EVs is more sensitive than that in serum alone. These results suggest that miRNAs in EVs may provide a new tool in the diagnosis of non-invasive liver cancer.

MiR-21 is an important marker identified in several studies of liver diseases. Numerous studies have shown that miR-21 of circulating EVs acts as a biomarker in glioblastoma multiform, colon, liver, and ovarian cancers (Taylor and Gercel-Taylor, 2008; Guo X. et al., 2017; Masoudi et al., 2017; Tsukamoto et al., 2017). The expression of miR-21 is closely related to HCC. A recent research shows that circulating miR-21 serves as a serum biomarker for HCC (Guo X. et al., 2017), in which the results showed that HCC patients exhibited significantly higher serum levels of miR-21 than healthy donors (*P* < 0.0001). Besides, Wang et al. (2014) found that the concentration of miR-21 was significantly higher in EVs than in EV-depleted supernatants or the whole serum. Further, the expression level of serum EV miR-21 was significantly higher in patients with HCC than those with CHB or healthy volunteers (Wang et al., 2014). In this study, we found that the expression of hsa-miR-21-5p in EVs was significantly higher than that in EV-free serum. This indicates that miR-21-5p was enriched in EVs, which functions through EVs. Besides, the expression of EV miR-21-5p in patients with CHB was higher than that in healthy donors. With the development of CHB to HCC, the expression of EV miR-21-5p in patients with HCC was reduced compared with CHB patients but still higher than healthy donors. A study also showed that serum miR-21 in patients with chronic hepatitis was higher than that in patients with HCC (Xu et al., 2011), which is consistent with our findings. The expression of miR-21-5p in EVs changed with the development of HCC, which is the innovation of this study.

In addition, miR-144 plays an important role as a tumor suppressor in many cancers including HCC. A few researches revealed that miR-144 suppresses the growth, proliferation, and metastasis of HCC by different signaling pathways (Yu et al., 2016; Bao et al., 2017; He et al., 2017; Liang et al., 2017), while, few people have studied the role of miR-144 of serum EVs in HCC. In this study, we first found that miR-144-3p was significantly enriched in EVs, and EV miR-144-3p was up-regulated in patients with HCC compared with healthy donors and patients with CHB. However, miR-144-3p does not necessarily function as a tumor suppressor gene in different tumors. For example, Sharma et al. (2016) found that significant upregulation of miR-144 in 29 of 35 (83%) esophageal cancer tissues as compared to matched distant non-malignant tissues (*P* = 0.010). In this study, we also found that the expression of miR-21-5p and miR-144-3p in hepatocellular carcinoma (BOT) was significantly higher than that in the distal tissues of hepatic carcinoma (BON). This is consistent with the research trends of Sharma et al. (2016). We found that the difference of miR-144-3p expression may be related to autophagy. A research demonstrated that Mycobacterium bovis Bacillus Calmette-Guérin (BCG) infection of macrophages leads to increased expression of miR-144-3p, which targets autophagy-related gene 4a (ATG4a), to inhibit autophagy activation and antimicrobial responses to BCG (Guo L. et al., 2017). Besides, another study revealed a previously unrecognized role of human MIR144 in the inhibition of antibacterial autophagy and the innate host immune response to *Mycobacterium tuberculosis* (Mtb) (Kim et al., 2017). The above studies show that the expression of miR-144-3p may be related to the process of autophagy, which needs more follow-up researches to prove it.

We have already known that miR-21-5p and miR-144-3p were up-regulated in EVs of patients with HCC but the relationship between them is not yet clear. Therefore, we count the ratio of miR-144-3p/miR-21-5p to clarify the relationship between them. The results showed that the ratio of miR-144-3p/miR-21-5p in EVs of the three groups was higher than that in EV-free serum. Among them, the ratio of miR-144-3p/miR-21-5p of EVs in patients with CHB was significantly reduced compared with healthy donors group. Besides, the ratio of miR-144-3p/miR-21-5p was significantly increased in the HCC group compared with patients with CHB. Further, the ROC curve also showed that the miR-144-3p/miR-21-5p had greater performance than AFP in diagnosing HCC. Just like ALT/AST is commonly used as a biochemical indicator for the diagnosis of liver disease clinically, miR-144-3p/miR-21-5p in serum EVs may provide a method to evaluate liver disease. As we all know, the development of HCC is mainly from healthy individuals to CHB patients, and further develop into HCC. In this study, one thing we notice is that 19 patients were HBsAg positive among 24 HCC patients, so it may provide some information of HBV-related HCC. EV-associated hsa-miR-21-5p and hsa-miR-144-3p are markedly elevated in serum of patients with HCC, and the trend of the ratio of miR-144-3p/miR-21-5p correlated with the development of HCC. It showed some interesting information, and the potential role of these microRNAs in the pathogenesis of HCC is worth of further study.

## Data Availability

Technical appendix, statistical code, and dataset were available from the corresponding author at wufei0348@126.com and shaoshujuan2006@126.com. Participants gave informed consent for data sharing.

## Author Contributions

CP proposed the study. CP and HH performed partial research and wrote the first draft. ZW, QZ, ZZ, and YL do partial experiments. WZ and LQ collected and analyzed the data. FW and SS modified the manuscript and they were the guarantor.

## Conflict of Interest Statement

The authors declare that the research was conducted in the absence of any commercial or financial relationships that could be construed as a potential conflict of interest.
